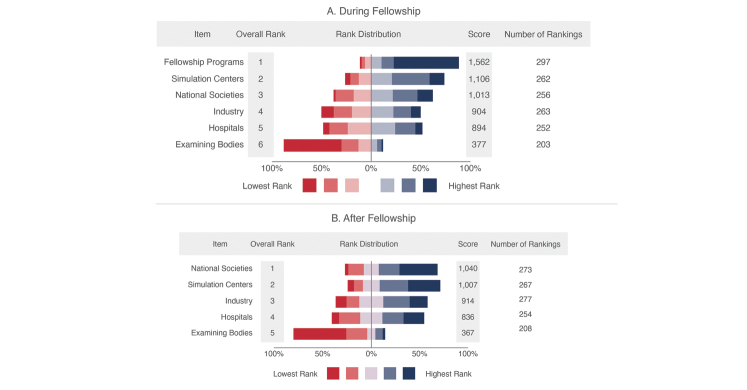# Current State of Simulation in Interventional Cardiology Training: Results of a SCAI Survey

**DOI:** 10.1016/j.jscai.2025.102566

**Published:** 2025-02-27

**Authors:** Kwan S. Lee, Andrew J. Klein, Rory S. Bricker, Arash Salavitabar, Nkechinyere N. Ijioma, Julia H. Indik, Sasanka N. Jayasuriya, F. David Fortuin, Abdulla A. Damluji, Emmanouil S. Brilakis, Dmitriy N. Feldman, Timothy D. Henry, John C. Messenger

**Affiliations:** a Department of Cardiovascular Medicine, Mayo Clinic Arizona, Phoenix, Arizona; b Department of Cardiovascular Medicine, Piedmont Heart, Atlanta, Georgia; c Division of Cardiology, University of Colorado School of Medicine, Aurora, Colorado; d Department of Pediatrics, Nationwide Children’s Hospital, The Ohio State University, Columbus, Ohio; e Division of Cardiovascular Disease, The Ohio State University, Columbus, Ohio; f Department of Cardiovascular Diseases, Mayo Clinic, Jacksonville, Florida; g Division of Cardiology, University of Arizona, Tucson, Arizona; h Department of Cardiovascular Medicine, Ascension Medical Group, Milwaukee, Wisconsin; i Inova Center of Outcomes Research, Inova Health, Fairfax, Virginia; j Department of Cardiovascular Medicine, Minneapolis Heart Institute, Minneapolis, Minnesota; k Department of Cardiovascular Medicine, Weill Cornell Medicine, New York, New York; l Department of Cardiovascular Medicine, The Christ Hospital, Cincinnati, Ohio

**Keywords:** education, interventional cardiology, safety, simulation

## Abstract

**Background:**

Interventional cardiology (IC) is well suited to simulation education, with a wide spectrum of digital and physical models for procedural training. Despite this, standardization, validation, and access to simulation training remains inconsistent in the United States and globally. Ten years have elapsed since the last Society for Cardiovascular Angiography & Interventions (SCAI) expert consensus statement on simulation in IC, which included a survey of US program directors. In this document, we report the results of a follow-up survey with the goal of broadening polling to all career stages, both in the US and internationally.

**Methods:**

A web-based 19-item survey with embedded subquestions was sent out via email solicitation to SCAI members from September 2023 to December 2023.

**Results:**

In total, 420 responses were collected, with a 15% response rate. Nearly 70% of respondents were from the US. There was equal distribution in responses for all stages of training, with most respondents performing coronary procedures. Two-thirds had previous exposure to simulation training with most using digital simulators and reporting only 1 to 2 days of exposure for each type or procedure. A majority (71%) felt that they had insufficient simulation training; most felt that simulation fidelity was average. The biggest barrier to simulation training was a lack of access.

**Conclusions:**

Despite efforts to develop simulation in IC training, there remain gaps in accessibility, exposure, and curricula. Professional organizations, industry, and educational governing bodies must collaborate on specific, actionable strategies to enhance access to high-fidelity IC simulation training globally.

## Introduction

Simulation has been widely used in health care practice and education as it standardizes the learner experience and ensures the development of competencies in a safe and structured environment without exposing patients to early learning curves. Interventional cardiology (IC) is well suited for simulation given the complexity and spectrum of procedures, rapid innovation requiring new techniques, steep learning curves, and the importance of developing competencies surrounding the recognition and appropriate response to infrequent but life-threatening complications. From a training perspective, simulation can help offset inconsistencies in procedural exposure given declining individual procedural volumes and work hour restrictions.^1^, ^2^, ^3^, ^4^, ^5^ The recent COVID-19 pandemic, with its profound impact on training, highlighted the importance and need for alternative models of non–patient-based training.^6^

The overall benefits of technology-enhanced simulation compared with those of traditional training in health care education has been clearly documented.^7^ Several observational and small randomized studies have demonstrated the benefits of using simulators for IC training. Simulators shorten the time to achieve competencies and improve trainee performance compared with traditional training methods^8^, ^9^, ^10^, ^11^ These studies have spanned coronary, peripheral, congenital, and structural intervention. In recognition of the benefits of simulation, both US and European cardiology IC training statements mandate trainee exposure to simulation both as a training tool and to evaluate competencies.^12^, ^13^, ^14^, ^15^, ^16^ The American Board of Internal Medicine attempted to incorporate simulation in maintenance of certification, but this has not seen widespread adoption.^17^

Despite recommendations by major professional societies and educational governing bodies that simulation be incorporated in training, there is a paucity of guidance on how exactly simulation should be used. Widespread adoption of simulation training remains hampered by their high cost, sparsity of expertise in simulation-based teaching, and lack of institutional financial support for time spent teaching or the cost of equipment. The optimal frequency of exposure, modality of simulation, and teacher-to-trainee ratios remain to be defined and will vary depending on the nature of the skills one is developing. Owing to these limitations, most procedural simulation training currently occurs at regional or national events on an infrequent basis and is heavily supported by medical industry, which has also focused on funding and development of procedural simulation specific to their portfolios.

A wide range of simulation task trainer technologies are currently in use for IC training. There are simple plastic-based, rubber-based, or silicone-based anatomical models such as those used as vascular access or vascular closure device models (dry simulators). Some models are fluid filled to simulate blood flow for vascular access (wet simulator without circulation), while others are significantly more complex, including pumps within the circuit, which generate pulsatility (wet simulator with circulation) such as those used in intracoronary physiology or complex percutaneous coronary intervention (PCI) training. The most sophisticated simulators currently available are digital haptic feedback platforms, which are used to simulate a wide spectrum of endovascular, coronary, and structural procedures. Although not widely available, the recent development of virtual and augmented reality modules presents new opportunities for educational innovation.^18^

Ten years have elapsed since the publication of the last Society for Cardiovascular Angiography & Interventions (SCAI) clinical document on procedural simulation in IC, which included a survey of US program directors.^19^ In order to update current exposure to and assess attitudes and opinions of the role of simulation in IC training, we conducted a survey of a wider audience, including both US and outside US (OUS) SCAI members, of all career stages.

## Methods

A 19-question online survey (Supplemental Material) was developed by members of SCAI and distributed to SCAI members (fellows and practicing and retired interventional and invasive cardiologists) from September 2023 to December 2023. Following an initial email solicitation, 3 reminder emails were sent. Additional survey responses were solicited via SCAI’s online member community platform, SCAI online newsletters, SCAI social media accounts, and word of mouth. Survey respondents were given the option to submit their contact information for a draw for free registration to SCAI’s annual scientific meeting. The survey (Appendix 1) included questions on demographics; previous simulation exposure (type, duration, location, and model); perceived fidelity of simulation; the types of procedures best suited for simulation training; barriers to simulation adoption; and opinions on leadership for simulation initiatives. An online self-service assessment tool (Alchemer) was used as the platform for the survey. A 5-point Likert scale was used to assess perceptions of simulator fidelity and utility. The resultant database collected the data hierarchically, such that both individual and group responses could be queried. This study was reviewed by the Mayo Clinic Institutional Review Board and deemed exempt from Institutional Review Board oversight. This article does not report on patients or patient data.

Descriptive data were obtained from survey responses. Data are presented as absolute number of responses for each choice and percentages of each choice/total.

## Results

The survey was successfully emailed to 2801 cardiologists with active SCAI membership. During the survey campaign (September 5, 2023, to December 19, 2023), 420 surveys were completed, corresponding to a response rate of 15%.

### Demographics

Respondent demographics are summarized in Table 1. Most (70%) respondents were from the US, 30% were from OUS with a wide distribution of countries (n = 37), 81% were men, and 38% were aged 20 to 39 years, 30% were aged 40 to 49 years, and 33% were older than 50 years; 11% were general cardiology fellows, 10% interventional/structural cardiology fellows, 17% were <5 years in practice, 16% were 5 to 10 years in practice, and 44% were >10 years in practice, with 2% retired. Most respondents performed coronary interventions (88%), followed by structural (42%), peripheral (35%), and congenital (31%) interventions, with the remainder being invasive, noninterventional cardiologists (16%).

**Table 1:** Respondent demographics and simulation exposure (United States and outside United States).

	All (N = 420)	United States (n = 292)	Outside United States (n = 128)
	%	%	%
Gender			
Male	81	80	85
Female	18	19	15
Age range (y)			
20-29	3	2	5
30-39	35	35	33
40-49	30	25	40
≥50	33	37	23
Stage of practice			
General cardiology fellow	11	13	8
Interventional cardiology fellow	10	8	16
<5 y in practice	17	18	16
5-10 y in practice	16	16	15
>10 y in practice	44	44	45
Retired	2	2	0
Type of practice			
Coronary	88	86	90
Structural	42	42	42
Peripheral	35	39	24
Adult congenital	21	18	29
Pediatric congenital	9	8	12
Invasive noninterventional	16	15	20
Previous simulation exposure			
Yes	67	72	56
No	33	28	44
Types of simulator			
Digital procedural	71	74	64
Virtual/augmented reality	29	26	38
Wet simulator with circulation pump	50	53	42
Wet simulator without circulation	45	43	48
Dry simulator model	60	64	47
Animal laboratory	40	47	20
Site of simulation training			
Simulation laboratory at my institution	49	55	34
Simulation laboratory at other institution	39	36	48
Local simulation course	25	20	39
Industry-organized training	78	79	73
National cardiology meetings	57	58	56
Type of training			
Self-directed	33	33	33
1-on-1 mentored	53	57	41
Small group (2-5) mentored	84	85	83
Large group (>5) mentored	31	26	47

### Overall exposure to simulation

**Figure 1:**
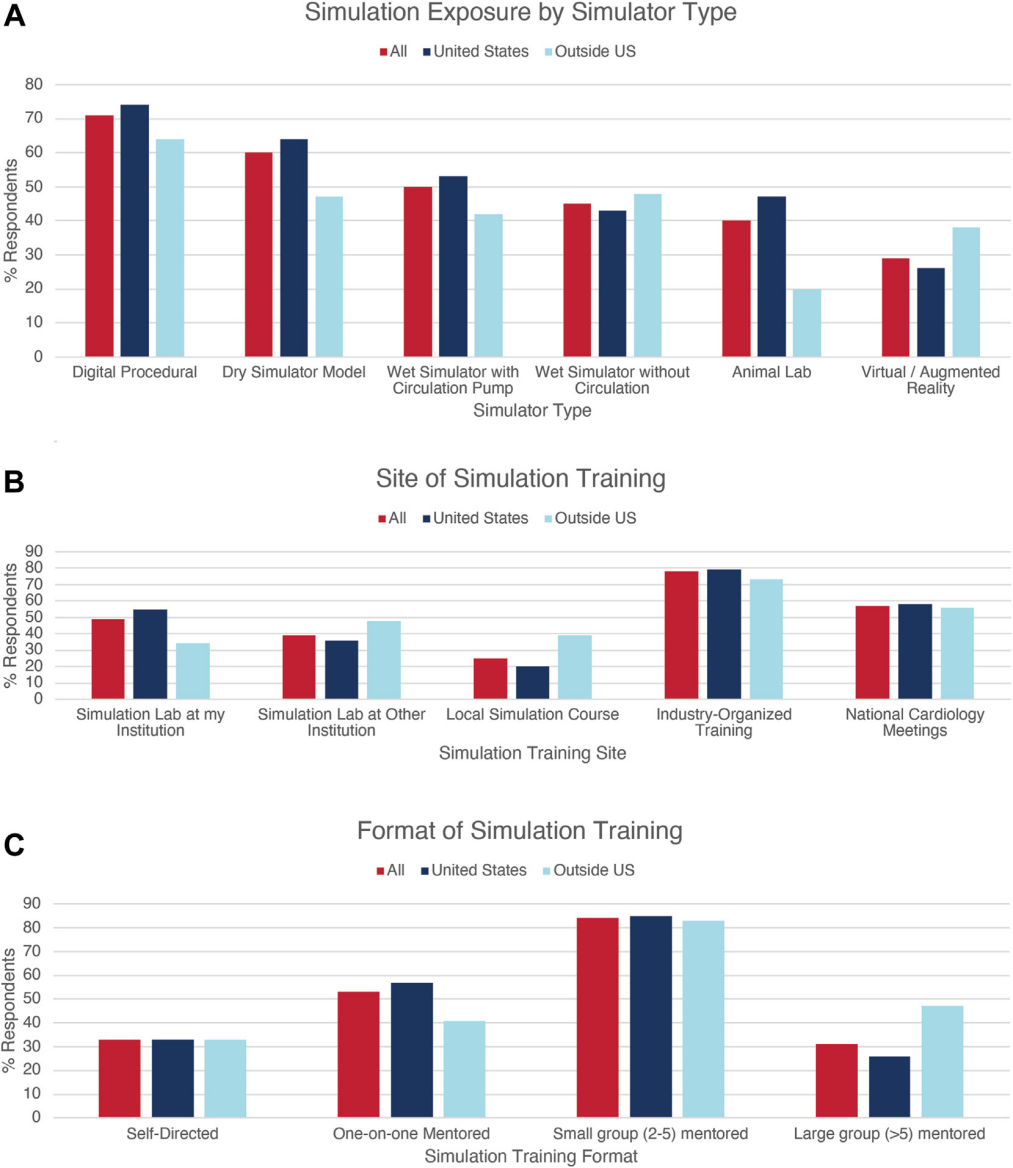
**Exposure by simulator type, site of training, and type of training; stratified by practice location.**

The respondents’ exposure to simulation is summarized in Table 1, Central Illustration, and Figure 1. Two-thirds (67%) of respondents had been exposed to IC simulation training, while 33% had no exposure (Central Illustration). Most respondents who had previously used simulators had used digital procedural simulators (71%), followed by dry simulators (cardiovascular model without fluid) (60%), a wet simulator with circulation pump (50%), a wet simulator without circulation (45%), animal laboratory exposure (40%), and, finally, a virtual/augmented reality simulator (29%) (Figure 1). The type and duration of specific simulation exposure is summarized in Table 2. The most common type of coronary procedure simulation was coronary angiography (73%) and basic PCI (65%). The most common type of structural procedure simulation was transcatheter valve replacement (TVR) (55%) and trans-septal puncture (37%). The lowest response rate was for pericardiocentesis, with only 18% of respondents receiving simulation training. Fifty-three percentage of respondents had echocardiography exposure through simulation, including transthoracic echocardiogram, transesophageal echocardiogram, and intracardiac echocardiogram. When present, most respondents reported limited exposure to simulation training, with only 1 to 2 days of simulation exposure for each type of simulation exposure. The number of participants reporting >1 week exposure to any specific type of simulation training was <10%, with a small minority of 1% of responders reporting >30 days of exposure for a specific procedure. Moreover, 78% of respondents experienced simulation training at industry-organized events, followed by 58% at national cardiology meetings, and 49% at simulation laboratories based at the home institution. Most training was mentored, in small groups of 2 to 5 trainees (84%), followed by mentored, 1-on-1 training (53%), self-directed (33%), and mentored in large groups (>5 trainees; 31%) (Figure 1).

**Central Illustration:**
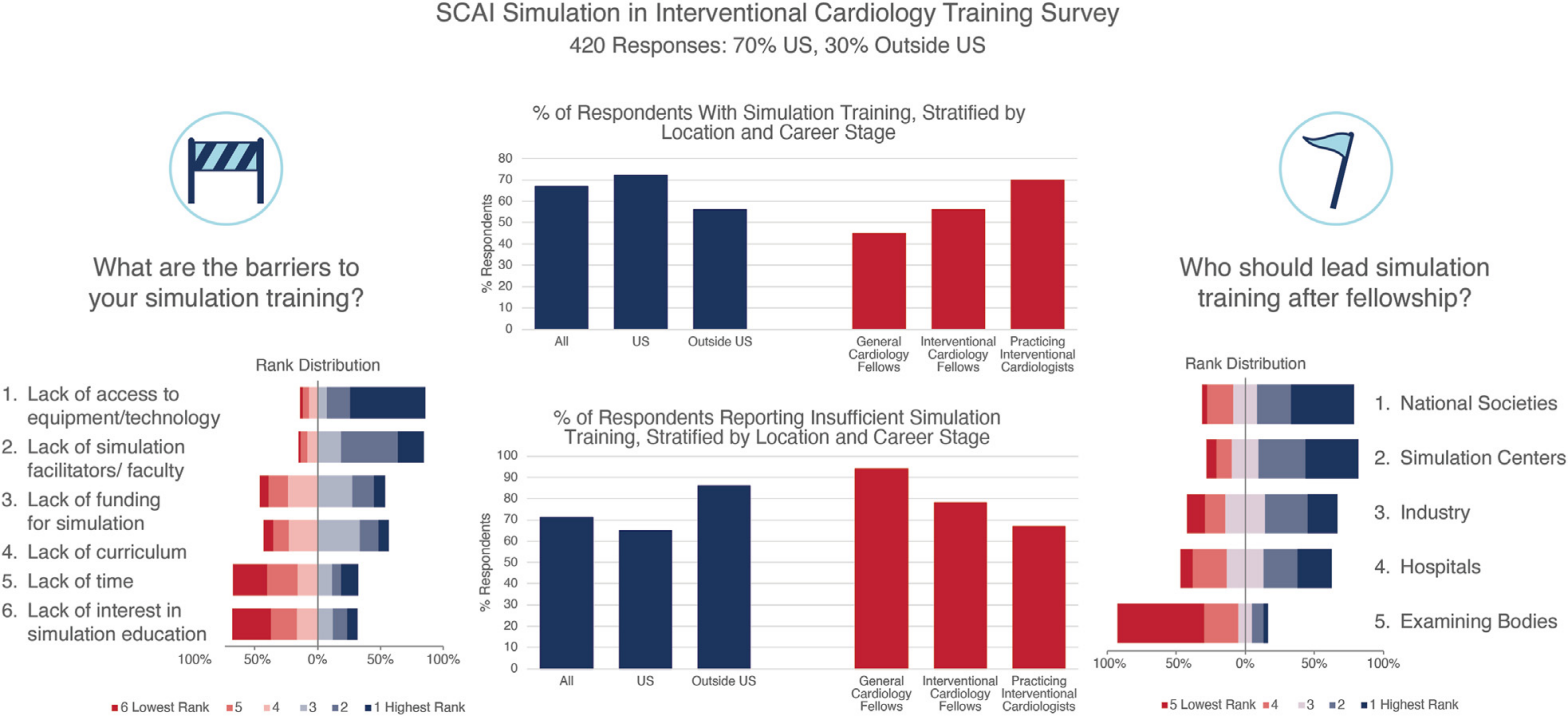
**Summary of the SCAI Simulation in Interventional Cardiology Training Survey.**

**Table 2:** Overall exposure to specific simulators by number of days, ranked by previous exposure

Procedure	No. of days					Total responses	Previous exposure (n)	% of total
	0	1-2	3-7	8-30	>30			
	(%)							
Coronary angiography	27	44	19	7	3	240	176	73
Basic PCI	35	45	14	3	2	237	153	65
Transcatheter valve replacement	46	34	15	5	1	231	126	55
Echocardiogram: TTE, TEE, ICE	47	34	15	2	1	226	119	53
Atherectomy	52	37	9	2	0	229	109	48
Intracoronary imaging/physiology	53	32	11	3	1	229	108	47
Trans-septal	54	36	6	3	1	290	106	37
Radial coronary angiography	55	29	11	3	3	232	105	45
Bifurcation PCI	61	26	8	2	2	228	88	39
PFO/ASD	64	24	9	2	1	232	83	36
TEER	69	20	7	4	0	309	72	23
LAAO	72	22	4	2	1	228	65	29
CTO	72	18	7	1	1	228	64	28
Peripheral intervention	72	16	8	1	2	225	63	28
Carotid stenting	78	17	4	1	0	227	50	22
Pericardiocentesis	82	11	3	3	1	224	41	18

ASD, atrial septal defect; CTO, chronic total occlusion; ICE, intracardiac echocardiogram; LAAO, left atrial appendage occlusion; PCI, percutaneous coronary intervention; PFO, patent foramen ovale; TEE, transesophageal echocardiogram; TEER, transcatheter edge-to-edge repair; TTE, transthoracic echocardiogram.

### Opinions on simulation

**Figure 2:**
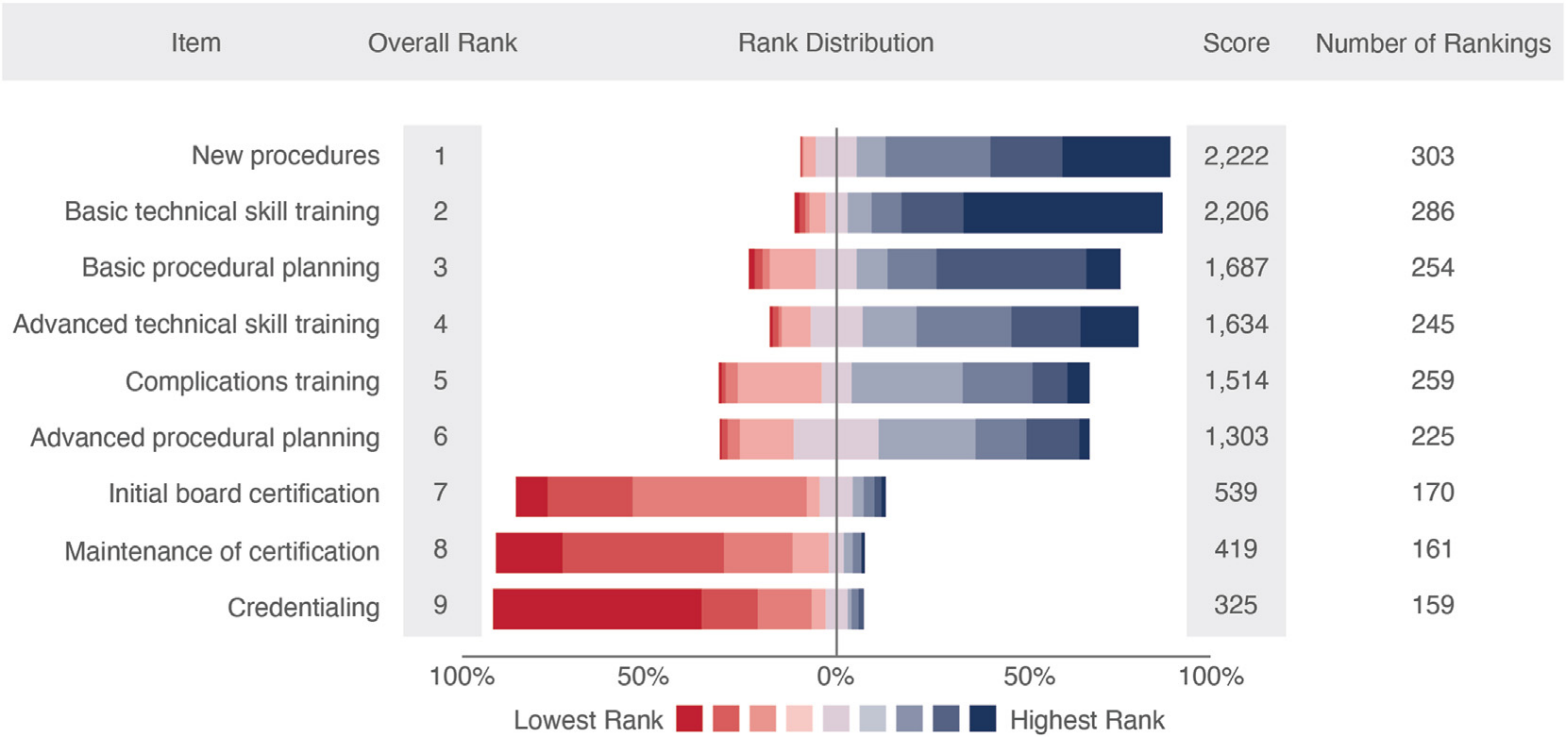
**What do you think simulation is most useful for and what are the barriers to simulation?**

**Supplementary Figure S1:**
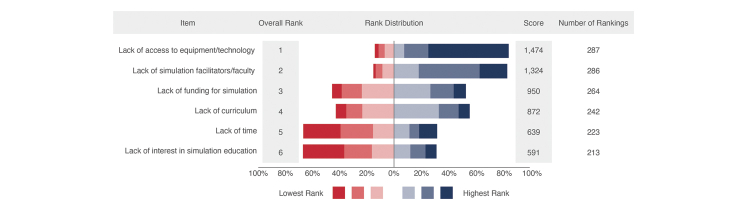


The respondents’ overall opinions on simulation training are summarized in Tables 3 and 4, Central Illustration, Figure 2, and Supplemental Figures S1 and S2. The majority (71%) of respondents felt that they had not had enough simulation training. (Central Illustration) Nearly half of the respondents (47%) felt that the simulators were of average fidelity (3/5), with a very small proportion (3%) reporting feeling they were completely life-like (5/5) and a small proportion (6%) reporting the opposite, not at all life-like (1/5). Respondents ranked simulation most useful for training in new procedures, followed by technical skill training and basic procedural planning. In contrast, simulation was ranked as least useful for credentialing, maintenance of certification, and initial board certification (Figure 2). Overall, respondents rated simulation as very helpful for trans-septal puncture, transcatheter edge-to-edge repair (TEER), patent foramen ovale (PFO)/ASD closure, TVR, left atrial appendage occlusion (LAAO), intracoronary imaging/physiology, echocardiography, and bifurcation PCI. In contrast, simulation was felt to be least helpful for pericardiocentesis, radial coronary angiography, and chronic total occlusion PCI. Most (84%) respondents rated simulation usefulness for specific procedures, between 3 and 5 (of 5) (Table 4). The largest barriers to simulation training were felt to be lack of access to equipment/technology, lack of simulation facilitators or faculty, and a lack of funding (Central Illustration and Supplemental Figure S1). Most respondents felt that during fellowship, simulation training should be led by fellowship programs, simulation centers, and national societies. After fellowship training, most respondents felt that simulation training should be led by national societies, simulation centers, and industry (Central Illustration and Supplemental Figure S2). However, the central role of industry was further reflected in the 93% “yes” response to the question “Should industry be responsible for providing simulation training for new procedures/techniques?”

**Table 3:** Overall Opinions on Simulation with US and outside US breakdown

	Response	All (N = 322) (%)	United States (n = 223) (%)	Outside United States (n = 99) (%)
Do you feel you have had enough simulation training?	Yes	29	35	14
Should industry be responsible for providing simulation training for new procedures?	Yes	93	92	96
How close to reality are the simulators you train with? (1 = not at all life-like; 5 = completely life-like)	1	6	7	3
	2	24	27	16
	3	47	47	46
	4	20	19	25
	5	3	1	10

**Table 4:** Opinions on how helpful specific simulators were for learning (1: not at all helpful, 5: very helpful) ranked by overall average

Procedure	1	2	3	4	5	Total responses	Average score
	(%)						
Trans-septal puncture	3	5	22	26	44	291	4.0
TEER	3	6	33	37	42	269	4.0
PFO/ASD	2	6	27	31	34	290	3.9
TAVR	2	4	27	30	37	289	3.9
LAAO	3	5	25	29	38	278	3.9
Intracoronary imaging/physiology	3	11	25	25	36	298	3.8
Echocardiogram: TTE, TEE, ICE	4	9	23	26	38	290	3.8
Bifurcation PCI	7	9	24	23	37	291	3.8
Atherectomy	5	13	23	29	30	292	3.7
Basic PCI	5	11	32	25	27	301	3.6
Coronary angiography	8	14	26	23	29	309	3.5
Carotid stenting	6	11	32	28	24	273	3.5
Peripheral intervention	5	13	33	26	23	248	3.5
CTO	10	15	25	21	30	288	3.4
Radial coronary angiography	11	16	28	23	23	304	3.3
Pericardiocentesis	11	18	25	21	26	284	3.3

ASD, atrial septal defect; CTO, chronic total occlusion; ICE, intracardiac echocardiogram; LAAO, left atrial appendage occlusion; PCI, percutaneous coronary intervention; PFO, patent foramen ovale; TAVR, transcatheter aortic valve replacement; TEE, transesophageal echocardiogram; TEER, transcatheter edge-to-edge repair; TTE, transthoracic echocardiogram.

### Opinions by career stage

**Figure 3:**
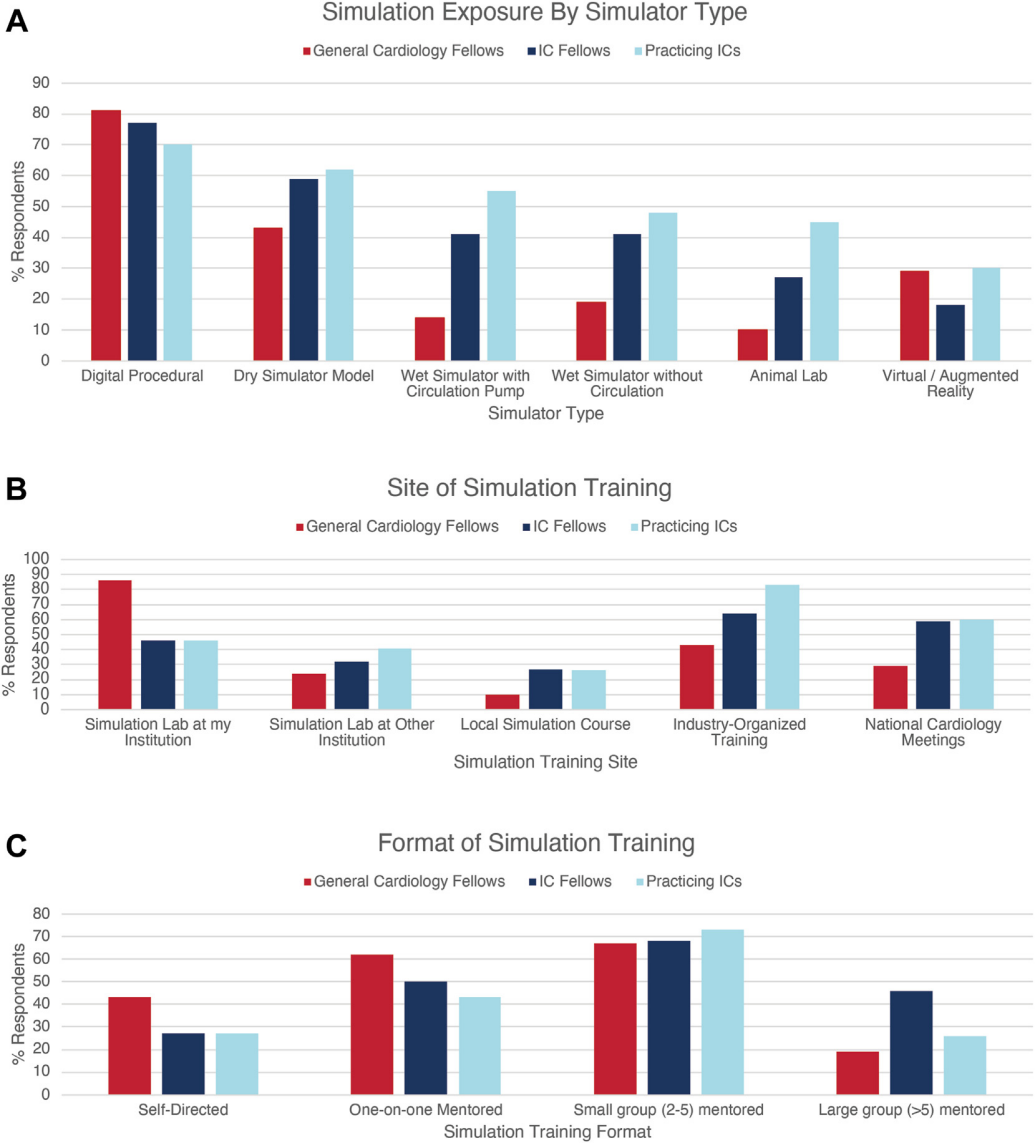
**Prior simulation exposure by simulator type and site of training, stratified by career stage.** IC, interventional cardiology.

Women represented 25% of cardiology fellow respondents and 16% of practicing IC respondents. As shown in Supplemental Table S1, general cardiology fellows were less likely to have been exposed to simulators compared with IC fellows or practicing interventional cardiologists (45% vs 56% vs 72%, respectively); however, insufficient simulation training was reported by respondents across all career stages (94% of general cardiology fellows, 78% of IC fellows, and 67% of practicing interventional cardiologists) (Central Illustration). Simulation exposure by type, site, and format of training is summarized in Figure 3. Similar to practicing interventional cardiologists, most cardiology fellows had used digital procedural simulators and dry simulators, while practicing interventional cardiologists were more likely to have been to an animal laboratory compared with fellows. IC fellows were the most likely to have been exposed to advanced procedural simulators compared with practicing interventional cardiologists and general cardiology fellows. General cardiology fellows’ exposure was mostly at a basic procedural level. Proportionally, more practicing interventional cardiologists were exposed to structural procedure simulation compared with fellows. General cardiology fellows were far more likely to have been exposed to simulation training at their home institution compared with IC fellows and practicing interventional cardiologists (86% vs 46% vs 46%), while practicing interventional cardiologists were far more likely to have been exposed to industry-organized training (83% vs 64% for IC fellows and 43% for general cardiology fellows). Sixty percentage of both IC fellows and practicing interventional cardiologists had been exposed to simulation training at national cardiology meetings, 43% of general cardiology fellows had self-directed simulation training, and 88% of practicing interventional cardiologists had small group mentored training. Fellows were more likely to respond that simulators were closer to reality than practicing interventional cardiologists. Fellows (general cardiology and IC) (Supplemental Table S2) and practicing interventional cardiologists agreed that the main barriers to simulation training were lack of access to equipment/technology and lack of simulation facilities. Similar to practicing interventional cardiologists, fellows were least likely to see a role for simulation in the maintenance of certification, credentialing, and initial board certification. Unlike practicing interventional cardiologists, fellows were more likely to feel that their fellowship programs, simulation centers, or their hospitals were responsible for leading simulation training during fellowship training. Practicing interventional cardiologists felt that national societies were most important in leading simulation training after fellowship. Overall, examination boards/bodies were universally felt to have the smallest role in leading simulation training.

### United States vs international responses

Overall, 30% of survey responses were from OUS with equivalent completion rates of 80%. Thirty-seven countries from all major geographical regions were represented, with responses from Asia (n = 45), Australia and Pacific Islands (n = 4), Europe (n = 27), the Middle East (n = 6), South America (n = 2), Africa (n = 10), and Canada/Mexico (n = 4). The highest response counts were from Vietnam (n = 23), India (n = 16), and Pakistan (n = 10). Respondents from OUS reported similar proportions of coronary and structural practice, with lower prevalence of peripheral and higher of congenital proceduralists (Table 1). The exposure to simulation was lower OUS, with 56% of interventionalists reporting having used simulation before compared with 72% in the United States. As shown in Figure 1, OUS simulator exposure was mostly with digital procedural simulators, both dry and wet simulator models, with lower exposure to animal laboratory experiences. When present, simulation exposure was almost entirely <7 days in duration among both US and OUS respondents. As in the US simulation exposure, most OUS simulation exposure was at industry-organized training and national cardiology meetings. However, OUS exposure was reported at non–home institution–based simulation laboratories more frequently than among US-based counterparts. In general, OUS practitioners responded more favorably when asked how close to reality they felt simulators were (Table 3). US responders were more likely to feel that they had enough exposure to simulation training (35% vs 14%). Opinions on barriers to simulation and who should lead simulation training during fellowship were similar, but OUS responders were more in favor of simulation centers and hospitals leading simulation training after fellowship, while US responders were much more likely to list industry as the lead.

## Discussion

Our survey represents the largest professional society-lead survey of IC professional opinions on the current state of simulation education to date, which lays the critical groundwork for the future advancement of simulation training for IC. Despite the development of a wide spectrum of devices and models for procedural simulation education over the last 2 decades, much work remains to organize, validate, and effectively disseminate simulation into standardized curricula within the life-long learning paradigm. This survey highlights a substantial unmet need for simulation training with 71% of respondents indicating inadequate exposure and 33% reporting no previous experience. Most reported experiences were brief, typically under a week, highlighting the need for further investigation into the optimal duration and frequency of simulation training.

It is also important to recognize that the overall fidelity of simulators is currently moderate and that the fiscal and technological barriers to creating high-fidelity simulation make the attainment of life-like fidelity unlikely. Therefore, we must work to identify the specific competencies that can be taught by simulator training and focus on these, with integration of such training into validated standard curricula. Our survey has identified specific procedures in which simulation training is more beneficial, especially for structural/congenital training (trans-septal puncture, TVR, TEER, LAAO, and PFO/atrial septal defect closure) and complex coronary procedures. These observations may help in developing best practices for simulation training including method of delivery, venue, frequency of exposure, and confirmation of skill acquisition. We need to work on supporting funding and providing resources for professional trainer development at all levels.

The survey confirms varying simulation needs across career stages. For example, general fellows benefit from foundational procedural training, but practicing interventional cardiologists may require simulation for new and advanced procedures in the field. This highlights the need for tailored simulation programs that address evolving learning requirements from early training through advanced practice. It is widely agreed that industry should help to support the funding and development of simulation education associated with the introduction of novel technology. Despite possible biases and risks of unbalanced representation, the track record of industry in supporting such efforts thus far has been good, with 78% of responses indicating exposure at industry-sponsored events, coupled with positive reviews of simulation education with procedures, which have seen large-scale global rollouts, such as TVR, LAAO, PFO closure, and TEER. Our survey appears to also identify a paucity of modules and content for congenital cardiologists and highlights the need for further development focused on this group. As this survey further shows, there appears to be an expectation and desire that professional societies and local hospitals assume a leading role in simulation training. It is therefore imperative that more resources, organizational rigor, and focus on the part of our national professional societies be paid to offering simulation training and developing validated content. Consensus guidelines and best practices for simulation education should be developed, preferably via a Delphi approach in order to begin to identify a consensus on content, modality, venue, and duration of simulation training. In contrast, the use of simulation for credentialing and during initial and re-certification is unpopular and likely premature for the current state of development.

### Study limitations

This study has several limitations, primarily related to the biases inherent to the voluntary, solicited nature of the survey. With a 15% response rate among SCAI members, we cannot be certain that the perspectives of all interventional cardiologists have been captured.^20^ Nonetheless, our study’s response rate is similar to other published survey studies of the SCAI membership, using similar methodology, with response rates of 10% to 15%.^6^^,^^21^, ^22^, ^23^, ^24^, ^25^ Reassuringly, the proportion of respondents was well distributed, with nearly equal representation of fellows vs early-career, mid-career, and late-career interventionalists. Given the wide global practice variation and experience, the aggregate responses of international interventional cardiologists are at best an approximation of global opinions, although it is worth noting that the responses for the most part were consistent with US responses.

## Conclusions

Simulation training holds the promise of facilitating patient safety and providing a safe environment for learners to receive IC training and education. Despite the advances in simulation technology, this contemporary survey demonstrated significant underutilization of this technology. Future efforts should focus on understanding which IC skills are best suited to a simulation learning environment and on fostering collaboration of all stakeholders to increase simulation training worldwide.

## Figures and Tables

**Supplementary Figure S2:**