# Supplementary material for: Current State of Simulation in Interventional Cardiology Training: Results of a SCAI Survey
Source: J Soc Cardiovasc Angiogr Interv. 2025 Feb 27;4(3Part A):102566. doi: 10.1016/j.jscai.2025.102566 (PMC11993868; doi:10.1016/j.jscai.2025.102566)
Supplement: Supplementary Material [file mmc1.docx]

**SUPPLEMENTAL TABLE 1: Respondent Demographics & Simulation Exposure by Career Stage (General, IC Fellow and Non-Fellows)**

|  |  | All (n=420) | General Fellow (n=47) | IC Fellow (n=43) | Non-Fellow (n=330) |
| --- | --- | --- | --- | --- | --- |
|  |  | % | % | % | % |
| Gender | Male | 81 | 72 | 77 | 83 |
|  | Female | 18 | 28 | 21 | 16 |
| Type of Practice | Coronary | 88 | 80 | 84 | 70 |
|  | Structural | 42 | 21 | 56 | 43 |
|  | Peripheral | 35 | 16 | 42 | 36 |
|  | Adult Congenital | 21 | 16 | 21 | 22 |
|  | Pediatric Congenital | 9 | 5 | 19 | 9 |
|  | Invasive Non-Interventional | 16 | 41 | 16 | 13 |
| Prior Simulation Exposure | Yes | 67 | 45 | 56 | 72 |
| Types of Simulator | Digital Procedural | 71 | 81 | 77 | 70 |
|  | Virtual / Augmented Reality | 29 | 29 | 18 | 30 |
|  | Wet Simulator with Circulation Pump | 50 | 14 | 41 | 55 |
|  | Wet Simulator without Circulation | 45 | 19 | 41 | 48 |
|  | Dry Simulator Model | 60 | 43 | 59 | 62 |
|  | Animal Lab | 40 | 10 | 27 | 45 |
| Site of Simulation Training | Simulation Lab at My Institution | 49 | 86 | 46 | 46 |
|  | Simulation Lab at Other Institution | 39 | 24 | 32 | 41 |
|  | Local Simulation Course | 25 | 10 | 27 | 26 |
|  | Industry-Organized Training | 78 | 43 | 64 | 83 |
|  | National Cardiology Meetings | 57 | 29 | 59 | 60 |
| Type of Training | Self-Directed | 33 | 43 | 27 | 33 |
|  | One-on-one Mentored | 53 | 62 | 50 | 52 |
|  | Small group (2-5) mentored | 84 | 67 | 68 | 88 |
|  | Large group (>5) mentored | 31 | 19 | 46 | 31 |

**SUPPLEMENTAL TABLE 2: Overall Opinions on Simulation by Career Stage**

|  |  | General Fellow (n=36) | IC Fellow (n=32) | Non-Fellow (n=254) |
| --- | --- | --- | --- | --- |
|  |  | % | % | % |
| Do you feel you have had enough simulation training? | Yes | 6 | 22 | 33 |
| Should industry be responsible for providing simulation training for new procedures? | Yes | 91 | 94 | 94 |
| How close to reality are the simulators you train with? (1=Not at all life-like; 5=Completely life-like | 1 | 0 | 0 | 7 |
|  | 2 | 24 | 18 | 24 |
|  | 3 | 43 | 50 | 47 |
|  | 4 | 33 | 23 | 19 |
|  | 5 | 0 | 9 | 3 |

**SUPPLEMENTAL FIGURE LEGENDS:**

**SUPPLEMENTAL FIGURE 1: What are the barriers to your simulation training?**

**SUPPLEMENTAL FIGURE 2: Who do you feel should lead simulation training A) during and B) after cardiology fellowship?**
