# Supplementary material for: Current State of Simulation in Interventional Cardiology Training: Results of a SCAI Survey
Source: J Soc Cardiovasc Angiogr Interv. 2025 Feb 27;4(3Part A):102566. doi: 10.1016/j.jscai.2025.102566 (PMC11993868; doi:10.1016/j.jscai.2025.102566)
Supplement: Supplementary Material [file mmc2.docx]

# Appendix 1: Simulation in Interventional Cardiology Training Survey

# Simulation in Interventional Cardiology Training Survey

## PAGE 1

## Please complete this short survey to help us understand the past, present and future role of simulation in interventional cardiology training. At the completion of the survey, you will have the option to provide your contact information to be entered into a raffle for 1 of 2 free registrations for the 2024 SCAI Scientific Sessions (May 2-4, 2024, in Long Beach, California).

#### 1) Where is your practice located? *

#### 2) What is your gender?

( ) Female

( ) Male

( ) Other

( ) Prefer not to answer

#### 3) What is your age range?

( ) 20-29

( ) 30-39

( ) 40-49

( ) >= 50

#### 4) What stage of training/practice are you in cardiology?

( ) 1st year general cardiology fellow

( ) 2nd year general cardiology fellow

( ) 3rd year general cardiology fellow

( ) Interventional/Structural cardiology fellow

( ) <5 years in Practice

( ) 5-10 years in Practice

( ) >10 years in Practice

( ) Retired

#### 5) What type(s) of procedural cardiology do you practice? (Select all that apply)

[ ] Coronary

[ ] Structural

[ ] Peripheral

[ ] Adult Congenital

[ ] Pediatric Congenital

[ ] Invasive non-interventional

#### 6) Have you used simulation-based training in cardiology before?*

( ) Yes

( ) No

**Page logic: IF:** #6 Question "Have you used simulation-based training in cardiology before?" is "No" **THEN:** Jump to PAGE 3

## PAGE 2

#### 7) If yes, which kinds of simulators have you used? Select all that apply.

[ ] Digital procedural simulator

[ ] Virtual / Augmented Reality simulator

[ ] Wet simulator with circulation pump (fluid-filled cardiovascular model)

[ ] Wet simulator without circulation (fluid-filled cardiovascular model)

[ ] Dry Simulator (cardiovascular model without fluid)

[ ] Animal lab

#### 8) What kinds of procedures and how many days of exposure have you had for simulation training?

|  | **None** | **1-2 days** | **3-7 days** | **8-30 days** | **>30 days** |
| --- | --- | --- | --- | --- | --- |
| Coronary angiography | ( ) | ( ) | ( ) | ( ) | ( ) |
| Radial coronary angiography | ( ) | ( ) | ( ) | ( ) | ( ) |
| Basic Percutaneous Coronary Intervention | ( ) | ( ) | ( ) | ( ) | ( ) |
| Intracoronary imaging / physiology | ( ) | ( ) | ( ) | ( ) | ( ) |
| Atherectomy | ( ) | ( ) | ( ) | ( ) | ( ) |
| Chronic Total Occlusion (CTO) | ( ) | ( ) | ( ) | ( ) | ( ) |
| Bifurcation | ( ) | ( ) | ( ) | ( ) | ( ) |
| Trans-septal puncture | ( ) | ( ) | ( ) | ( ) | ( ) |
| Left atrial appendage occlusion | ( ) | ( ) | ( ) | ( ) | ( ) |
| Transcatheter Valve Replacement | ( ) | ( ) | ( ) | ( ) | ( ) |
| Transcatheter Edge-to-Edge Repair (TEER) | ( ) | ( ) | ( ) | ( ) | ( ) |
| Patent Foramen Ovale (PFO)/ Atrial Septal Defect (ASD) Closure | ( ) | ( ) | ( ) | ( ) | ( ) |
| Pericardiocentesis | ( ) | ( ) | ( ) | ( ) | ( ) |
| Echocardiography, Intra-cardiac echocardiography (ICE), Transesophageal echocardiography (TEE) | ( ) | ( ) | ( ) | ( ) | ( ) |
| Carotid Artery Stenting | ( ) | ( ) | ( ) | ( ) | ( ) |
| Peripheral Intervention | ( ) | ( ) | ( ) | ( ) | ( ) |
| Other (please enter) | ( ) | ( ) | ( ) | ( ) | ( ) |

#### 9) Where have you been exposed to simulation training? Select all that apply

[ ] Simulation lab based at my institution

[ ] Simulation lab based at other institution

[ ] Local simulation course

[ ] Industry-organized training

[ ] National cardiology meetings

#### 10) What type of simulation training have you been exposed to? Select all that apply

[ ] Self-directed

[ ] One-on-one mentored

[ ] Small group (2-5 trainees) mentored

[ ] Large group (>= 5 trainees) mentored

#### 11) On a scale of 1-5, how close to reality are the simulators you train with? (1= Not at all life-like; 5 = completely life-like)

Not at all life-like ( ) 1 ( ) 2 ( ) 3 ( ) 4 ( ) 5 Completely life-like

## PAGE 3

#### 12) Do you feel you have had enough simulation training?

( ) Yes

( ) No

#### 13) What do you think simulation is most useful for? Please rank items in order from most useful (top/1) to least useful (bottom/9).

________Basic technical skill training

________Basic procedural planning

________Advanced technical skill training

________Advanced procedural planning

________New procedures

________Complications training

________Initial board certification

________Maintenance of certification

________Credentialing

#### 14) Using a scale from 1 (not at all helpful) to 5 (very helpful), how helpful do you think simulation is for learning the following procedures?

|  | [ ] 1 (not at all helpful)  [ ] 2  [ ] 3  [ ] 4  [ ] 5 (very helpful) |
| --- | --- |
| Coronary angiography |  |
| Radial coronary angiography |  |
| Basic Percutaneous Coronary Intervention |  |
| Intracoronary imaging / physiology |  |
| Atherectomy |  |
| Chronic Total Occlusion (CTO) |  |
| Bifurcation |  |
| Trans-septal puncture |  |
| Left atrial appendage occlusion |  |
| Transcatheter Valve Replacement |  |
| Transcatheter Edge-to-Edge Repair (TEER) |  |
| Patent Foramen Ovale (PFO) / Atrial Septal Defect (ASD) Closure |  |
| Pericardiocentesis |  |
| Echocardiography, Intra-cardiac echocardiography (ICE), Transesophageal echocardiography (TEE) |  |
| Carotid Artery Stenting |  |
| Peripheral Intervention |  |

#### 15) What are the barriers to your simulation training? Please rank items in order from biggest barrier (top/1) to lowest barrier (bottom/6).

________Lack of access to equipment/ technology

________Lack of simulation facilitators/ faculty

________Lack of curriculum

________Lack of interest in simulation education

________Lack of funding for simulation

________Lack of time

16) Who do you feel should lead simulation training during cardiology fellowship? Please rank items in order from largest role (top/1) to smallest role (bottom/6). 
________Fellowship Programs

________Simulation Centers

#### ________Medical Schools

________Hospitals

________Industry

________National Societies

________Examining Bodies

#### 17) Who do you feel should lead simulation training after fellowship training? Please rank items in order from largest role (top/1) to smallest role (bottom/5).

________Fellowship Programs

________Simulation Centers

________Medical Schools

________Hospitals

________Industry

________National Societies

________Examining Bodies

#### 18) Should industry be responsible for providing simulation training for new procedures/techniques?

( ) Yes

( ) No

### 19) Please share any additional comments you have about simulation in cardiology training

## Thank you!
